# Enhancing Chemotherapy‐Related Immune Responses via Bioorthogonal Metabolic Engineering‐Driven Tumor Exosomes Elimination

**DOI:** 10.1002/advs.202506409

**Published:** 2025-06-11

**Authors:** Wentao Zhang, Tianyi Yang, Tian Jin, Tianyi Zhu, Fang Hao, Miao Fan, Yanrong Zhang

**Affiliations:** ^1^ Department of Thyroid and Breast Surgery The Second Hospital of Hebei Medical University Shijiazhuang 050000 China; ^2^ College of Pharmacy Hebei Medical University Shijiazhuang 050017 China; ^3^ Department of Pharmacology Hebei Medical University Shijiazhuang 050017 China; ^4^ Clinical laboratory The Third Hospital of Hebei Medical University Shijiazhuang 050000 China; ^5^ Department of Vascular Surgery The Third Hospital of Hebei Medical University Shijiazhuang 050000 China

**Keywords:** bioorthogonal metabolic engineering, chemotherapy, immunotherapy, phagocytosis, tumor exosomes

## Abstract

Tumor cell‐driven exosomes (TExo) have exhibited several major drawbacks that hinder antitumor therapy. A representative immunosuppressive mechanism is the depletion of CD8+ cytotoxic T cells with the help of exosomal PD‐L1. Another common mechanism is to promote tumor metastasis by promoting the seeding and growth of metastatic cancer cells in distant organs. Therefore, the removal of TExo can provide many benefits for the treatment of cancer patients. Here, a **bio**orthogonal **r**eaction‐**d**riven **e**xosome **e**limination (Biordee) strategy that promoted macrophage‐mediated phagocytosis by using IgG Fc to engineer endogenous TExo (TExo‐Fc) was developed. The Biordee strategy effectively reduced the levels of TExo in the circulatory system by leveraging the interaction of IgG Fc with FcγRII/III receptors of macrophage, which further broke down the body's immunosuppression and enhanced the immune response after chemotherapy. Moreover, the Biordee strategy inhibited breast cancer liver metastases, which were enhanced by promoting chemotherapy‐induced TExo release. This work provided a new attempt to reduce TExo level after chemotherapy to enhance antitumor therapeutic effects.

## Graphical abstract

In this work, a novel strategy named **bio**orthogonal **r**eaction‐**d**riven **e**xosome **e**limination (Biordee) strategy was developed to inhibit chemotherapy‐associated breast cancer metastasis and enhance the immune response. Biordee possessed the capacity of in‐situ editing tumor‐derived exosomes (TExo) with IgG Fc and promoted macrophage phagocytosis of engineered TExo. Biordee simultaneously blocked premetastatic effect of TExo and immunosuppressive effects of exosomal PD‐L1.

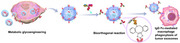

## 1 Introduction

Exosomes are a class of extracellular vesicles rich in bioactive substances (e.g., proteins, DNA, RNA, etc.) that play a key role in cell‐to‐cell communication by delivering bioactive substances into recipient cells.^[^ ^1^, ^2^ ^]^ Tumor cell‐driven exosomes (TExo), as a class of exosomes secreted by special cells, have been extensively studied and confirmed to play an important role in tumor progression and antitumor therapy.^[^ ^3^, ^4^ ^]^ TExo can preferentially seek out specific organs and build nests there, so as to prepare a favorable microenvironment for metastatic cancer cells in distant organs, thereby promoting tumor metastasis.^[^ ^5^, ^6^ ^]^ Furthermore, exosomal PD‐L1 can induce CD8+ cytotoxic T cells to develop a state of “fatigue” before reaching the tumor site, thereby inhibiting the systemic immune response and promoting tumor growth.^[^ ^7^, ^8^ ^]^ Therefore, the removal of endogenous TExo is considered to be an effective therapeutic pathway that can inhibit tumor metastasis and enhance antitumor immune responses. Drug inhibitors, such as GW4869 and sulfisoxazole, had been reported to be able to enhance antitumor effects by inhibiting TExo biogenesis.^[^ ^9^, ^10^ ^]^ Strategies based on the mechanism of exosome biogenesis were helpless against TExo that have entered the circulatory system. Therefore, some artificial intelligence nanomaterials had been developed to selectively bind and remove TExo.^[^ ^11^, ^12^ ^]^ For example, Jia et al. used aptamer (anti‐EGFR)‐modified mesoporous silicon nanoparticles to tow and dump circulating TExo from the blood into the small intestine.^[^ ^12^ ^]^ However, the application effect of artificial intelligence nanomaterials was limited by the positive rate of aptamers on TExo.

**Figure 1:**
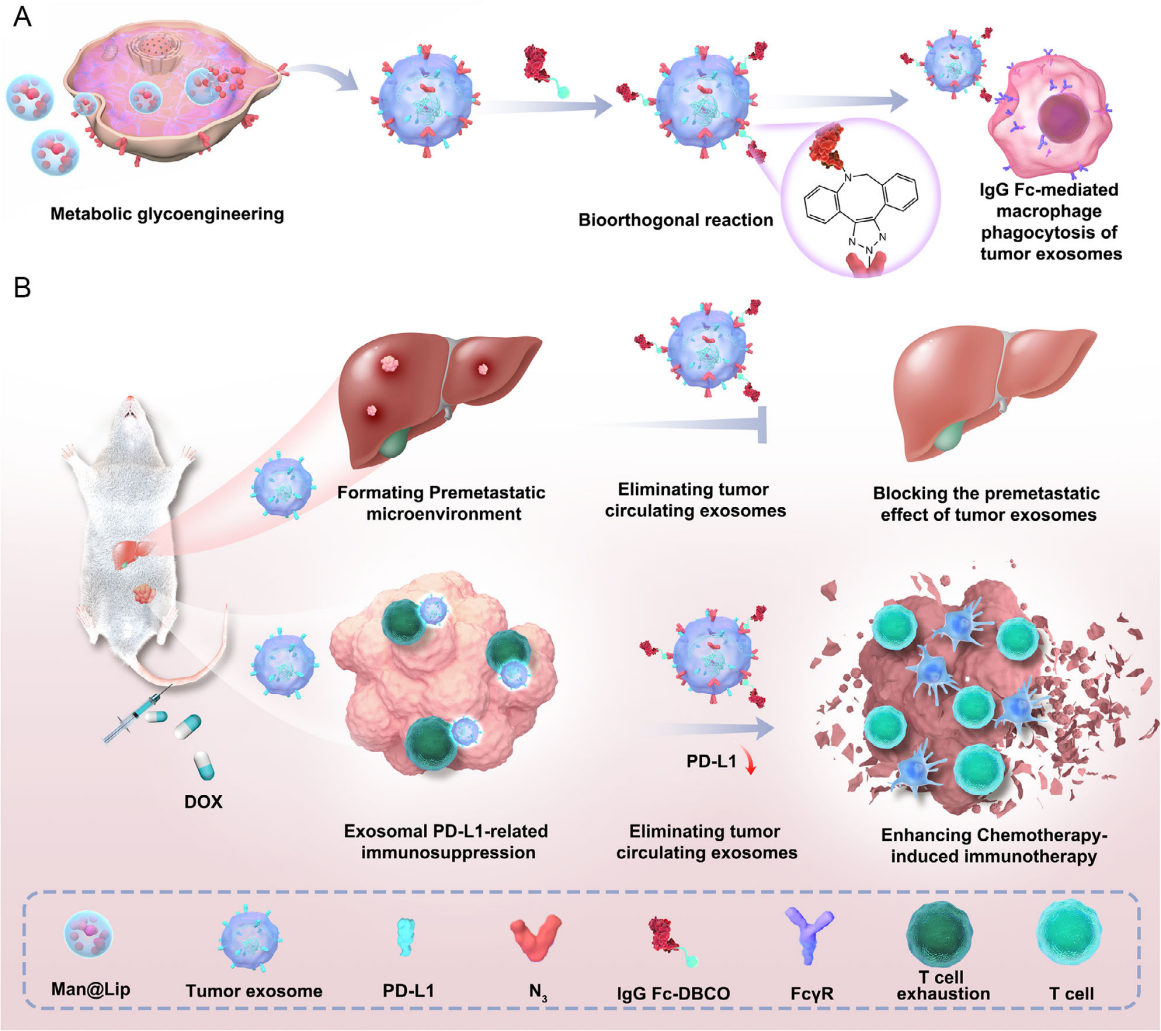
Schematic of clearance of TExo by Biordee strategy to inhibit tumor metastasis and enhance antitumor immune response. A) Schematic illustration of the construction of TExo‐Fc to promote macrophage phagocytosis using glycometabolic engineering and bioorthogonal reactions. B) Schematic illustration of the utilizing Biordee strategy to effectively inhibit chemotherapy‐associated breast cancer metastasis and enhance the immune response after chemotherapy.

At present, chemotherapy is still the first‐line clinical treatment for cancer. However, we noted recent studies suggesting that chemotherapy drugs (taxanes and anthracyclines) could trigger increased release of TExo, which in turn promoted cancer metastasis.^[^ ^13^, ^14^ ^]^ The results of this study suggested that the removal of TExo in cancer patients undergoing chemotherapy was undoubtedly necessary. Here, we developed a **bio**orthogonal **r**eaction‐**d**riven **e**xosome **e**limination (Biordee) strategy to inhibit chemotherapy‐associated breast cancer metastasis and enhance the immune response after chemotherapy (**Figure** **1**). Nonnatural sugars (such as mannose‐N_3_) were often used to label chemical tags (azido groups, N_3_) on cell membrane through glycometabolic engineering, which can be coupled to other molecules through efficient chemical reactions to achieve subsequent target molecular modification.^[^ ^15^ ^]^ Liposomes lacking engineered targeting groups, frequently employed as drug carriers, were recognized for their good biocompatibility and remarkable tendency to accumulate in tumor tissue.^[^ ^16^, ^17^ ^]^ We first constructed mannose‐N_3_@liposomes (Man@Lip) formulation to improve the feasibility of the Biordee strategy in future clinical applications. On this basis, glycometabolic engineering was used to label tumor cells to obtain TExo carrying N_3_ groups (TExo‐N_3_), mainly due to the presence of protein components derived from cell membranes on the exosomes.^[^ ^18^, ^19^, ^20^ ^]^ The Fc region of immunoglobulin G (IgG Fc) was selected as a modifier. The dibenzocyclooctyne group (DBCO)‐modified IgG Fc (IgG Fc‐DBCO) underwent a specific bioorthogonal reaction with TExo‐N_3_ to obtain IgG Fc‐engineered TExo (TExo‐Fc).^[^ ^21^, ^22^ ^]^ The interaction of IgG Fc on the surface of TExo‐Fc with the FcγRII/III receptor of macrophages promoted the phagocytosis of TExo by macrophages (Figure 1A).^[^ ^23^ ^]^ In this study, we established a mouse model of the chemotherapy drug doxorubicin (DOX) for the treatment of breast cancer. The results suggested that our Biordee strategy inhibited chemotherapy‐enhanced breast cancer liver metastases, and this enhanced metastasis was considered to be strongly associated with increased DOX‐induced TExo release. In addition, removal of endogenous TExo reduced the inhibition of the body's immune response by exosomal PD‐L1 and further enhanced the chemotherapy‐related immunotherapy (Figure 1B). Our Biordee strategy opened up a potential and feasible avenue to improve treatment efficacy and prognosis for chemotherapy patients.

## 2 Results

### 2.1 Construction and Characterization of TExo‐Fc

**Figure 2:**
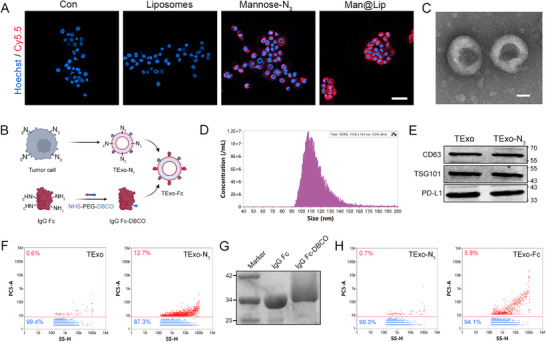
Construction and characterization of TExo‐Fc. A) CLSM imaging of N_3_ groups in 4T1 cell after 24 h of co‐incubation with PBS, liposomes, mannose‐N_3_, and Man@Lip. Scale bar, 50 µm. B) Scheme of the construction of TExo‐Fc. C) Representative TEM image of TExo‐N_3_. Scale bar, 50 nm. D) NTA result of TExo‐N_3_. E) Western blot analysis of TExo and TExo‐N_3_. F) Nano‐flow cytometry results of TExo and TExo‐N_3_. G) Coomassie blue staining analysis of the conjugation of DBCO‐modified IgG Fc. H) Nano‐flow cytometry results of TExo‐N_3_ and TExo‐Fc.

In order to improve the utilization rate of mannose‐N_3_, liposomes with a particle size of ≈150 nm were first synthesized (Figure S1, Supporting Information). Subsequently, mannose‐N_3_ was loaded into liposomes by electroporation to obtain a liposome delivery system (Man@Lip).^[^ ^24^ ^]^ The loading efficiency of mannose‐N_3_ in liposome was determined by UV‐Vis spectrophotometry, and the result showed that the loading efficiency was ≈60.3% (Figure S2, Supporting Information). The results of MTT assay showed that the liposome delivery system had no effect on the proliferation of 4T1 cells (Figure S3, Supporting Information). Flow cytometry analysis showed a strong fluorescence signal, indicating that 4T1 cells were efficiently uptaking Man@Lip (Figure S4, Supporting Information). On this basis, we explored the glucose metabolism engineering of Man@Lip in 4T1 cells. After 24 h of co‐incubation of 4T1 cells with Man@Lip, 4T1 cell membranes were labeled by DBCO‐CY5.5. The result of confocal laser scanning microscopy (CLSM) confirmed the presence of N_3_ groups on the surface of 4T1 cells (**Figure** **2**A). The result of Figure 2A also suggested that metabolic labeling of Man@Lip was mainly due to the action of mannose‐N_3_.

Subsequently, the exosomes secreted by the azide glycosaccharide‐treated 4T1 cells were extracted for further construction of TExo‐Fc (Figure 2B). Transmission electron microscopy (TEM) result showed that TExo‐N_3_ was a spherical vesicle structure with a particle size of ≈100 nm (Figure 2C; Figure S5, Supporting Information). The result of nanoparticle tracking analysis (NTA) was consistent with this (Figure 2D). Western blot results confirmed that TExo had exosomal marker proteins and an immunosuppressive protein PD‐L1 (Figure 2E). Next, the N_3_ group on the surface of TExo was labeled by DBCO‐CY5.5. As shown in Figure 2F, the positive rate of TExo‐N_3_ was significantly higher than that of TExo, indicating the presence of N_3_ groups on the surface of TExo‐N_3_. According to the fluorescence quantification method, each TExo carried ≈3400 N_3_ groups (Figure S6, Supporting Information).^[^ ^25^ ^]^ IgG Fc‐DBCO was ready for use in the construction of TExo‐Fc. Coomassie blue staining result showed that IgG Fc‐DBCO ran more slowly than IgG Fc, and that IgG Fc‐DBCO resulted in scattered bands on the gel (Figure 2G).^[^ ^26^ ^]^ Subsequently, TExo‐N_3_ was engineered using IgG Fc/FITC‐DBCO. The result of nanoflow cytometer showed an enhancement of the fluorescence signal, indicating that IgG Fc/FITC was successfully modified on the surface of TExo‐N_3_ through a bioorthogonal reaction (Figure 2H).^[^ ^27^ ^]^ These results confirmed the feasibility of constructing TExo‐Fc by glucose metabolism engineering and bioorthogonal reaction.

### 2.2 IgG Fc Promoted Phagocytosis of TExo by Macrophages

**Figure 3:**
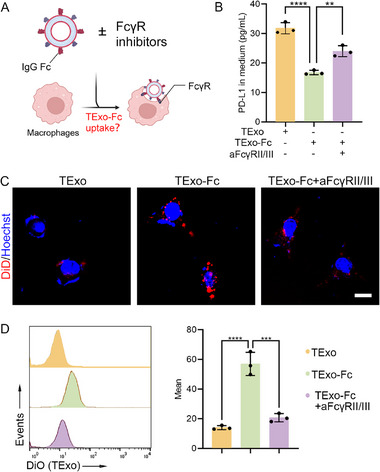
IgG Fc promoted phagocytosis of TExo by macrophages. A) Scheme of macrophages phagocytosis of TExo‐Fc via FcγR receptors. B) ELISA analysis of exosomal PD‐L1 levels in medium after 24 h of co‐incubation with macrophages (*n* = 3). C) CLSM imaging of macrophage phagocytosis of TExo‐Fc. Scale bar, 20 µm. D) Flow cytometry analysis and quantitative result of macrophage phagocytosis of TExo‐Fc (*n* = 3). Data are presented as the mean ± SD. ^*^ *p* < 0.05; ^**^ *p* < 0.01; ^***^ *p* < 0.001; and ^****^ *p* < 0.0001.

Next, we determined the binding kinetics of IgG Fc to FcγRII/III protein through a surface plasmon resonance (SPR) system. The SPR results showed that IgG Fc had a strong binding effect with FcγRIIB protein and FcγRIII protein (Figure S7, Supporting Information). On this basis, we explored the phagocytosis of TExo‐Fc by macrophages (**Figure** **3**A). After macrophages were co‐incubated with TExo‐Fc for 24 h, the content of exosomal PD‐L1 in medium was measured by ELISA. As shown in Figure 3B, macrophage phagocytosis of TExo‐Fc was significantly increased compared to TExo. However, the addition of anti‐FcγRII/III to the co‐culture system reduced the uptake of TExo‐Fc by macrophages, suggesting that macrophage phagocytosis of TExo‐Fc was through the interaction of IgG Fc with FcγRII/III receptors. CLSM images and flow cytometry analysis similarly supported this conclusion. (Figure 3C,D).

### 2.3 Elimination of Endogenous TExo Inhibited Tumor Growth

First, we gathered evidence from in vivo studies to validate the feasibility of constructing TExo‐Fc through bioorthogonal reactions. After 4 h of intravenous injection of IgG Fc/FITC‐DBCO, exosomes in peripheral blood were collected and used for fluorescence quantification. The significantly enhanced green fluorescence on the surface of exosomes confirmed the successful construction of Exo‐Fc (Figure S8, Supporting Information). The fluorescence changes in the IgG Fc/FITC‐DBCO group compared to the IgG Fc/FITC group suggested that the binding of IgG Fc to exosomes relied on biological orthogonal reactions. Next, we evaluated whether bioorthogonal reactions preferentially occurred on tumor‐derived rather than normal cell‐derived exosomes. DBCO‐CY5.5 was used to label the main organs of mice. The result of immunofluorescence staining showed that red fluorescence was observed only in tumor tissue after intratumoral injection of Man@Lip, indicating that the major organs were not modified with azide group (Figure S9, Supporting Information). However, N_3_ group‐labeled exosomes were detected in the major organs and peripheral blood of mice (Figure S10, Supporting Information). Fluorescence quantitative result of anti‐EGFR‐coated microplates indicated that N_3_ group‐labeled exosomes were TExo, implying that N_3_ group‐labeled TExo spread from tumor tissue to the whole body (Figure S11, Supporting Information). On this basis, exosomes in peripheral blood were specifically adsorbed by anti‐PD‐L1‐coated microplate and fluorescently quantified by microplate readers. As shown in Figure S12 (Supporting Information), the green fluorescence was concentrated in the microplate rather than the supernatant after incubation, confirming that bioorthogonal reactions occured primarily in TExo. To avoid the effects of PD‐L1 on nontumor cell‐derived exosomes, we reinforced this conclusion with anti‐EGFR‐coated microplates (Figure S12, Supporting Information).^[^ ^12^ ^]^ We further considered the distribution of IgG Fc‐DBCO. After intravenous injection of IgG Fc/FITC‐DBCO, green fluorescence was observed in the major organs of mice (Figure S13, Supporting Information). This result implied that biochemical orthogonal response‐mediated endogenous TExo modifications may occur in any organ and peripheral blood. The TExo‐Fc/FITC and free IgG Fc/FITC‐DBCO was basically metabolized after 72 h (Figure S14, Supporting Information). Furthermore, we evaluated the safety of the Biordee strategy. The blood biochemical results showed that elimination of TExo did not cause significant organ toxicity (Figure S15A–C, Supporting Information). Analysis of coagulation‐related markers showed that the removal of TExo had no adverse effects on coagulation function, which somewhat dispelled our concerns about the accidental depletion of physiologically important exosomes, such as coagulation‐related exosomes (Figure S15D, Supporting Information).

**Figure 4:**
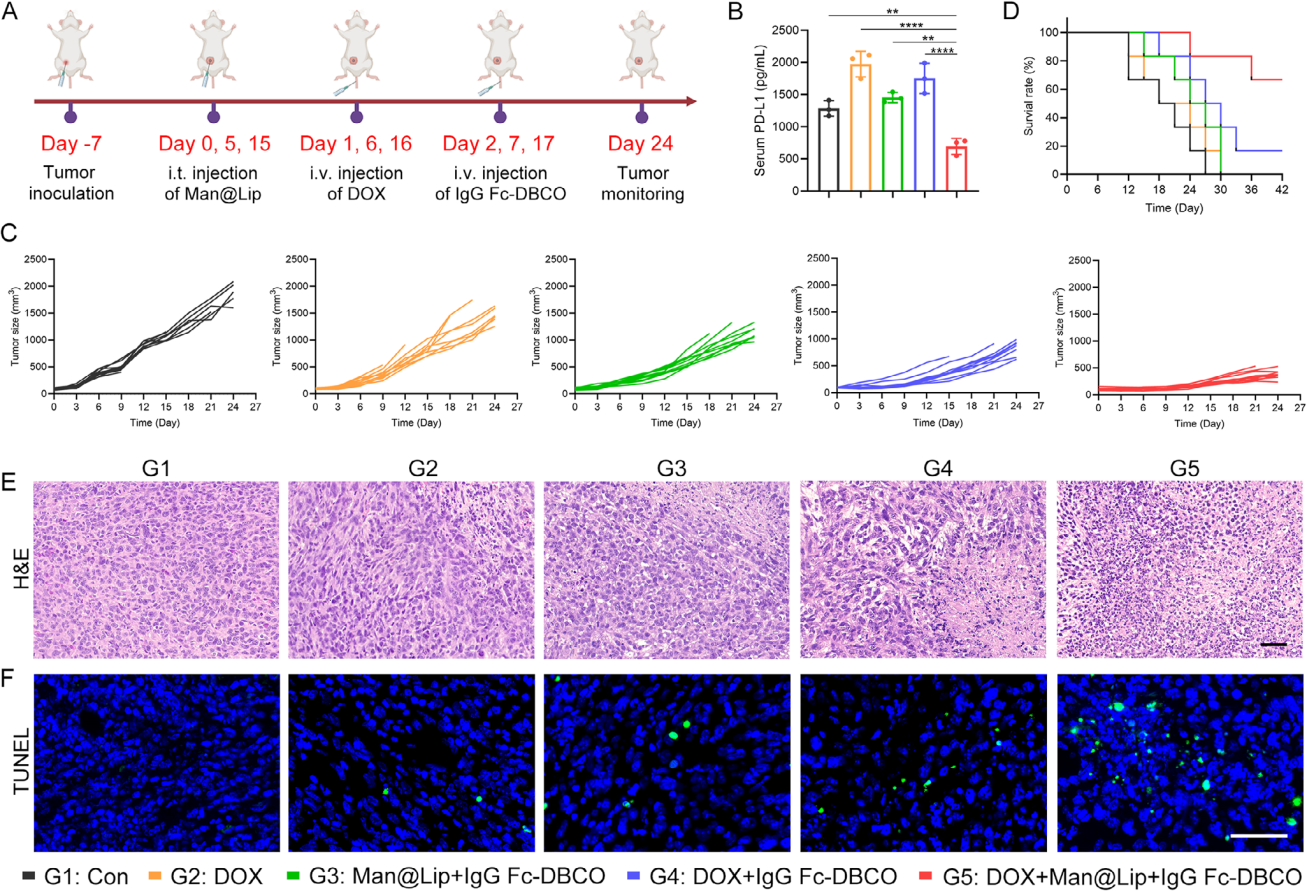
Elimination of endogenous TExo inhibited tumor growth. A) The schematic illustrated the use of the Biordee strategy in a tumor model of DOX for the treatment of breast cancer. B) ELISA analysis of exosomal PD‐L1 levels in peripheral blood (*n* = 3). C,D) Tumor growth curves (C) and survival curves (D) of mice in different treatment groups (*n* = 10). E,F) H&E (E) and TUNEL (F) staining images of tumor tissues in different treatment groups. Scale bar, 50 µm. Data are presented as the mean ± SD. ^*^ *p* < 0.05; ^**^ *p* < 0.01; ^***^ *p* < 0.001; and ^****^ *p* < 0.0001.

On this basis, we constructed a mouse model of DOX for breast cancer treatment to evaluate the effect of the Biordee strategy in enhancing anti‐tumor responses (**Figure** **4**A). We analyzed the levels of TExo in the peripheral blood of 4T1 tumor‐bearing mice. The result showed that the Biordee strategy significantly reduced the level of TExo in the peripheral blood of mice (Figure 4B). This result indirectly proved the feasibility of constructing TExo‐Fc in vivo through bioorthogonal reactions. In addition, we noted that DOX increased the number of TExo in the peripheral blood compared to the control group, suggesting that TExo clearance in chemotherapy patients was necessary and beneficial. We further evaluated the therapeutic effect of the Biordee strategy. Compared to the other treatment groups, G5 group significantly inhibited tumor growth (Figure 4C; Figure S16, Supporting Information). Body weight and survival curves showed that the Biordee strategy did not affect mouse body weight and significantly prolonged mouse survival (Figure 4D; Figure S17, Supporting Information). Moreover, H&E and TUNEL staining results showed that the combined application of DOX and Biordee strategy triggered more severe tissue necrosis and tumor cell apoptosis in the tumor tissues of mice (Figure 4E,F).

### 2.4 Elimination of Endogenous TExo Promoted Chemotherapy‐Related Immune Responses

**Figure 5:**
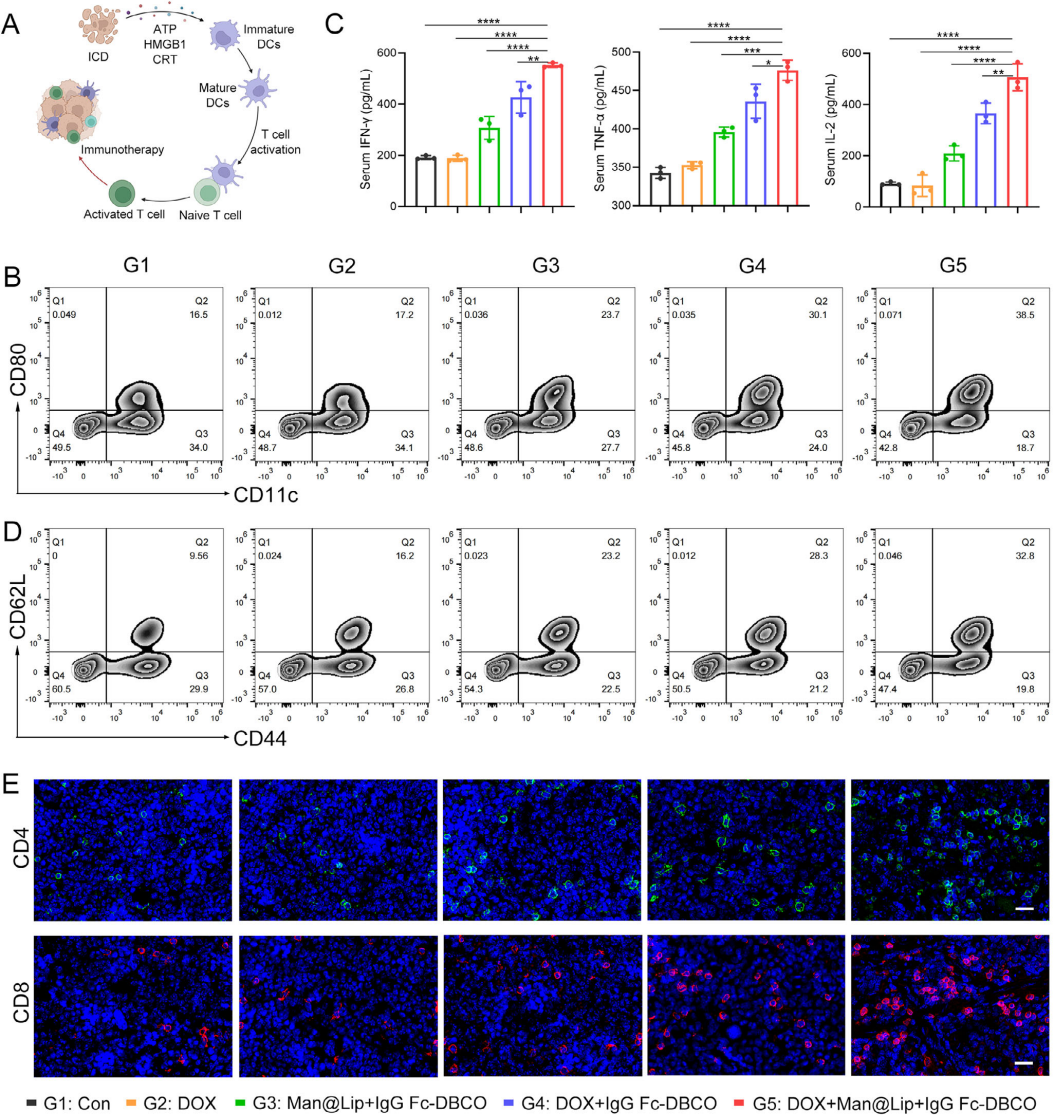
Elimination of endogenous TExo enhanced systemic antitumor immune response in mice. A) Scheme of DOX‐induced antitumor immune response. B) Flow cytometry analysis of CD11c+CD80+ cells in mouse spleen tissues from different treatment groups. C) ELISA analysis of the content of IFN‐γ, TNF‐α, IL‐2 in mouse serum (*n* = 3). D) Flow cytometry analysis of CD44+CD62L+ cells in mouse spleen tissues from different treatment groups. E) Immunofluorescence images of CD4+ and CD8+ T cells in spleen tissue. Scale bar, 20 µm. Data are presented as the mean ± SD. ^*^ *p* < 0.05; ^**^ *p* < 0.01; ^***^ *p* < 0.001; and ^****^ *p* < 0.0001.

Dox activated the body's immune response by inducing immunogenic cell death (ICD) of tumor cells (**Figure** **5**A; Figure S18, Supporting Information).^[^ ^28^, ^29^ ^]^ First, ICD effect and dendritic cell (DC) maturation was evaluated. Significant DC activation was present in all treatment groups (Figure 5B; Figures S19 and S20, Supporting Information). Among them, DC maturation in the treatment group containing DOX was due to the potent triggering of ICD production. DC maturation in the G3 group may occurred through IgG Fc‐mediated antibody‐dependent cell‐mediated phagocytosis (ADCP).^[^ ^30^ ^]^ Compared with the G4 group, the number of mature DC in the G5 group was further increased, indicating that TExo elimination increased the maturation rate of DC.^[^ ^31^ ^]^ DOX‐induced ICD and immunomodulatory effects of IgG Fc could trigger efficient secretion of immune cytokines including IFN‐γ, TNF‐α, and IL‐2 (Figure 5C). Activated DC induced subsequent tumor‐specific T cell expansion and activation.^[^ ^32^ ^]^ The immunosurveillance cells including CD4+ T cells, CD8+ T cells, and the memory T cells were upregulated (Figure 5D,E; Figures S19 and S20, Supporting Information). Compared to the G4 group, the elimination of TExo further strengthened the action of T cells. The increase in the number of CDD44+CD62L+ T cells helped to prevent tumor recurrence after ablation of the primary tumor (Figure 5D; Figures S19 and S20, Supporting Information).

**Figure 6:**
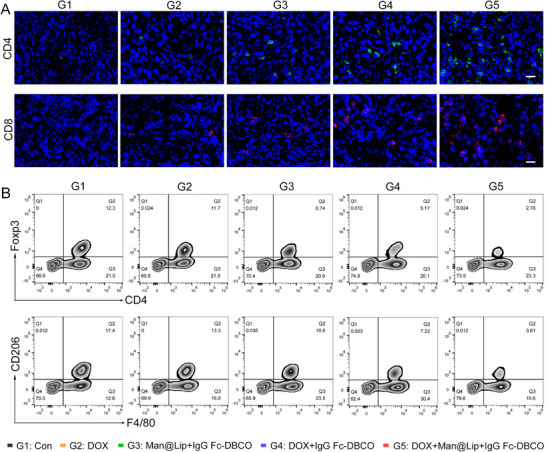
Elimination of endogenous TExo improved the immune microenvironment of tumor tissues. A) Immunofluorescence images of CD4+ and CD8+ T cells in tumor tissue. Scale bar, 20 µm. B) Flow cytometry analysis of CD4+Foxp3+ and F4/80+CD206+ cells in tumor tissues from different treatment groups.

The reduction of exosomal PD‐L1 in the circulatory system allowed T cell viability to be maintained until it reaches the tumor.^[^ ^7^ ^]^ Therefore, we further evaluated the effect of TExo clearance on the tumor immune microenvironment. Immunofluorescence staining confirmed that the Biordee strategy promoted antitumor T cell infiltration and activity (**Figure** **6**A). At the same time, the infiltration of immunosuppressive cell types, including CD4+Foxp3+ regulatory T cells and F4/80+CD206+ M2 macrophages, was effectively reduced (Figure 6B; Figures S19 and S21, Supporting Information). These immunological changes promoted the remodeling of the tumor immunosuppressive microenvironment and significantly inhibited breast cancer progression.^[^ ^33^ ^]^ Interestingly, we noted that IgG Fc‐DBCO that entered the tumor microenvironment bound not only to TExo, but also directly to the surface of tumor cells. IgG Fc‐DBCO, which entered the tumor microenvironment, bound to TExo and tumor cells with an efficiency of 38% and 54%, respectively (Figure S22, Supporting Information). This meant that IgG Fc could directly kill tumor cells by mediating antibody dependent cell‐mediated cytotoxicity (ADCC) or ADCP.^[^ ^30^ ^]^ Although IgG Fc‐DBCO was able to bind directly to tumor cells, IgG Fc‐DBCO that penetrated into the tumor microenvironment was a minority (Figure S13, Supporting Information). Thus, more IgG Fc was used to promote macrophage phagocytosis of TExo in nontumor tissues, rather than mediating ADCC by binding on the surface of tumor cells. In summary, in vivo studies had demonstrated that the synergistic effect of DOX and Biordee strategy was that the triggering of systemic immune responses by DOX‐induced ICD and FcγR activation, and that TExo clearance ensured the functional durability of activated T cells, thus maximizing immune effects. However, mechanistic studies of the intricate interactions between immune activation and TExo clearance remained limited. Future research will explore more deeply to develop more comprehensive therapeutic strategies to disrupt the complex immune evasion mechanisms of triple‐negative breast cancer.

### 2.5 Elimination of Endogenous TExo Inhibited Liver Metastasis of Breast Cancer

**Figure 7:**
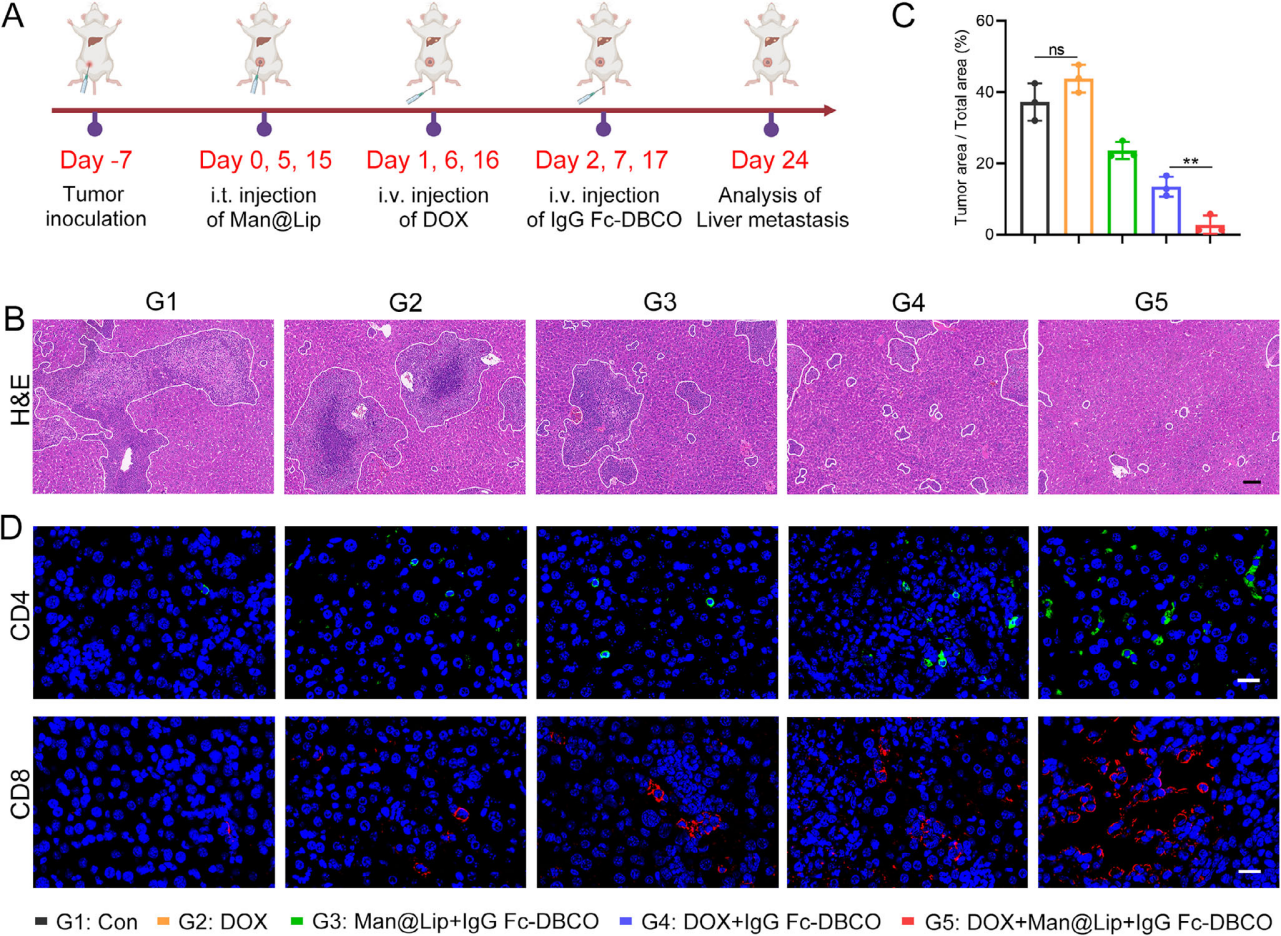
Elimination of endogenous TExo inhibited liver metastasis of breast cancer. A) The schematic illustrated the use of the Biordee strategy in a breast cancer liver metastasis model. B,C) H&E (B) staining images and quantitative results (C) of tumor tissue in different treatment groups (*n* = 3). Scale bar, 20 µm. D) Immunofluorescence images of CD4+ and CD8+ T cells in liver tissue. Scale bar, 20 µm. Data are presented as the mean ± SD. ^*^ *p* < 0.05; ^**^ *p* < 0.01; ^***^ *p* < 0.001; and ^****^ *p* < 0.0001.

Moreover, we evaluated the effect of the Biordee strategy in inhibiting tumor metastasis (**Figure** **7**A). As shown in Figure 7B,C, DOX induced an increasing trend toward breast cancer metastasis, which correlated with DOX‐facilitated TExo release. Removal of TExo effectively inhibited tumor metastasis. TUNEL staining result showed that the combination of DOX and Biordee strategy promoted apoptosis in metastatic tumor cells (Figure S23, Supporting Information). The infiltration of a large number of CD4+ and CD8+ T cells in liver tissue was also associated with inhibition of tumor metastasis, which was consistent with the previous conclusions (Figure 7D). Further, we validated the therapeutic efficacy of the Biordee strategy in breast cancer types with different PD‐L1 expression levels (Figure S24, Supporting Information). Although PD‐L1 expression in 231 cells was lower than that in 4T1 cells, we still observed that the Biordee strategy was able to effectively inhibit breast cancer liver metastases and lung metastases (Figure S25, Supporting Information). This result demonstrated the effectiveness and universality of the Biordee strategy. In conclusion, the Biordee strategy inhibited metastasis of breast cancer by blocking the pre‐metastatic effect of TExo and promoting immune cell infiltration.

## 3 Conclusion

In summary, we developed a Biordee strategy to remove TExo in vivo. First, we demonstrated that 4T1 cells labeled by glycometabolic engineering secrete N_3_‐carrying exosomes. In addition, IgG Fc could be bioconjugated to TuExo‐N_3_ via a bioorthogonal reaction. In vitro studies had shown that the interaction of IgG Fc on the surface of TExo‐Fc with the FcγRII/III receptor of macrophages promoted the phagocytosis of TExo by macrophages. Next, we constructed a mouse model of DOX for the treatment of breast cancer. In vivo studies had shown that DOX‐induced ICD and FcγR activation stimulated body immunity, while elimination of TExo could improve the immunosuppressive microenvironment and facilitate the preservation of T cell function. Under these three aspects, the growth of breast cancer tumors in situ was effectively inhibited. In addition, the Biordee strategy also favored the inhibition of chemotherapy‐promoted breast cancer liver metastases. All these results confirmed the feasibility and effectiveness of the Biordee strategy to enhance the efficacy of antitumor treatment. Although the Biordee strategy provided an innovative idea to improve the therapeutic effect of chemotherapy patients, there were still some limitations therein. For example, the complex internal environment of organisms may lead to differences in the efficiency of glycometabolic engineering of tumor cells and the labeling of endogenous exosomes. In addition, blood biochemistry was only a short‐term assessment of the in vivo safety of the Biordee strategy. In the future, research on the long‐term toxicity of the Biordee strategy, including the unintended depletion of physiologically important exosomes due to off‐target effects, should be intensified. At the same time, further study such as targeted delivery of Man@Lip, as well as the more validation of Biordee strategy in different tumor models, should also be investigated to facilitate a step toward clinical application.

## 4 Experimental Section

### Metabolic glyco‐engineering in 4T1 Cells

4T1 cells were seeded in 6‐well plates. After the cells were adhered, they were replaced with fresh medium containing DiO‐labeled Man@Lip (final Man concentration of 0.4 µM). After 24 h, the uptake of Man@Lip by 4T1 cells was detected by flow cytometry.

4T1 cells were seeded in confocal dishes. After the cells were adhered, they were replaced with fresh medium containing mannose‐N_3_, liposomes, and Man@Lip, respectively. After 24 h of continued culture, 4T1 cells were labeled with DBCO‐CY5.5 and the N_3_ group on the surface of the 4T1 cells was visualized by CLSM.

### Preparation of IgG‐Fc‐DBCO and TExo‐Fc

Mix the pre‐prepared NHS‐PEG‐DBCO (Xian Ruixi Biological Technology) solution and IgG Fc (Solarbio) solution (1:1 molar ratio) and shake the mixed solution on a shaker at 4 °C for 6 h.^[^ ^34^, ^35^ ^]^ Subsequently, the unreacted NHS‐PEG‐DBCO in solution was removed by dialysis, and the DBCO group‐modified IgG Fc (IgG Fc‐DBCO) was finally obtained.

The pre‐prepared IgG Fc‐DBCO solution was added to the purified TExo‐N_3_ solution and subsequently shaken at 4 °C for 1 h. Purified TExo‐Fc was obtained by ultracentrifugation to remove the unreacted IgG Fc‐DBCO from the solution.

### Macrophages Engulf TExo‐Fc

RAW264.7 cells were seeded in 6‐well plates. After the cells were adhered, they were replaced with fresh medium containing TExo, TExo‐Fc, and TExo‐Fc+anti‐FcγRII/III (Proteintech), respectively. After 24 h of continued culture, the content of TExo in the medium was measured by ELISA method. DiD‐labeled TExo and TExo‐Fc were used to detect TExo/TExo‐Fc uptake by RAW 264.7 cells by flow cytometry.

RAW264.7 cells were seeded in confocal dishes. After the cells were adhered, it was replaced with fresh medium containing DiD‐labeled TExo, TExo‐Fc and TExo‐Fc+anti‐FcγRII/III, respectively. After 24 h of continued culture, the uptake of TExo‐Fc by RAW 264.7 cells was observed by CLSM.

### The Biordee Strategy Enhances Chemotherapy‐Associated Immune Responses

All animal experiments were conducted according to the requirements of the Laboratory Animal Ethics and Welfare Committee of Hebei Medical University (Approval No. IACUC‐Hebmu 2 024 196). 5‐week age BALB/c mice were inoculated with 4T1 breast cancer cells in situ. When tumor volumes reach ≈80 mm^3^, mice randomly divided into five groups: PBS, DOX, Man@Lip+IgG Fc‐DBCO, DOX+IgG Fc‐DBCO and DOX+Man@Lip+IgG Fc‐DBCO. Mice in the PBS group were first intratumoral injection of 100 µL of PBS solution, the second intravenous injection of 100 µL of PBS solution over 24 h, and the third intravenous injection of 100 µL of PBS solution after 48 h. Mice in the DOX group were first intratumoral injection of 100 µL of PBS solution, the second intravenous injection of 100 µL of DOX solution over 24 h, and the third intravenous injection of 100 µL of PBS solution after 48 h. Mice in the Man@Lip+IgG Fc‐DBCO group were first intratumoral injection of 100 µL of Man@Lip solution, the second intravenous injection of 100 µL of PBS solution over 24 h, and the third intravenous injection of 100 µL of IgG Fc‐DBCO solution after 48 h. Mice in the DOX+IgG Fc‐DBCO group were first intratumoral injection of 100 µL of PBS solution, the second intravenous injection of 100 µL of DOX solution over 24 h, and the third intravenous injection of 100 µL of IgG Fc‐DBCO solution after 48 h. Mice in the DOX+Man@Lip+IgG Fc‐DBCO group were first intratumoral injection of 100 µL of Man@Lip solution, the second intravenous injection of 100 µL of DOX solution over 24 h, and the third intravenous injection of 100 µL of IgG Fc‐DBCO solution after 48 h. The treatment was repeated every 5 days for a total of three times. After the end of treatment, four mice were sacrificed in each group to collect tumor tissue and liver tissue. The collected tumor and liver tissue were used for further pathological and omics analysis. Mouse blood was collected, and the levels of cytokines were detected by ELISA kit (Bioss).^[^ ^36^ ^]^ The remaining mice were used to continue to observe the survival status and count the survival rate.^[^ ^37^, ^38^ ^]^

## Conflict of Interest

The authors declare no conflict of interest.

## Supporting information



Supporting Information

## Data Availability

Research data are not shared.
