# Supplementary material for: Enhancing Chemotherapy‐Related Immune Responses via Bioorthogonal Metabolic Engineering‐Driven Tumor Exosomes Elimination
Source: Adv Sci (Weinh). 2025 Jun 11;12(33):e06409. doi: 10.1002/advs.202506409 (PMC12412542; doi:10.1002/advs.202506409)
Supplement: Supplementary file 1 — Supporting Information [file ADVS-12-e06409-s001.docx]

Supporting Information

Enhancing Chemotherapy-Related Immune Responses via Bioorthogonal Metabolic Engineering-Driven Tumor Exosomes Elimination

*Wentao Zhang, Tianyi Yang, Tian Jin, Tianyi Zhu, Fang Hao, Miao Fan*, Yanrong Zhang**

W. T. Zhang, F. Hao

Department of Thyroid and Breast Surgery, The Second Hospital of Hebei Medical University, Shijiazhuang 050000, China

T. Y. Yang, Prof. M. Fan
College of Pharmacy, Hebei Medical University, Shijiazhuang 050017, China

E-mail: fanm0321@hebmu.edu.cn (M. Fan)

Prof. Y. R. Zhang

Department of Vascular Surgery, The Third Hospital of Hebei Medical University, Shijiazhuang 050000, China

1. mail: yanrongzhang@hebmu.edu.cn

T. Y. Zhu

Clinical laboratory, The Third Hospital of Hebei Medical University, Shijiazhuang 050000, China

T. Jin

Department of Pharmacology, Hebei Medical University, Shijiazhuang 050017, China

**Experimental**

**Synthesis of Man@Lip**

Weigh 20 mg of egg yolk lecithin and 10 mg of cholesterol and dissolve into a mixture of 3 mL of ethanol and 5 mL of chloroform. The mixed solution was transferred to a 25 mL round-bottom flask, where it was evaporated by rotation at 40 ℃. Add 20 mL of PBS solution to the flask containing the dried lipid membrane and form a translucent suspension with low-power sonication. Subsequently, liposomes with uniform particle size were obtained by means of a liposome extruder. The mannose-N_3_ solution was added to the pre-prepared liposome solution, and the mannose-N_3_ was subsequently loaded into the liposomes by electroporation. The excess mannose-N_3_ was removed by centrifugation, and finally purified Man@Lip were obtained. The loading efficiency of mannose-N_3_ was determined by establishing standard curves of concentration versus absorbance.

**Extraction and identification of TExo-N_3_**

4T1 cells were seeded in a cell culture dish. After the cells were adhered, replace with fresh medium containing Man@Lip. After 24 h, replace with serum-free medium. After 24 h of continued culture, the cell culture supernatant was collected for exosome extraction. The cell culture supernatant was centrifuged at 3,000 g for 10 min to remove dead cells and cell debris, followed by 10,000 g for 30 min to remove impurities. The supernatant was concentrated to a suitable volume by centrifugation at 4,500 g for 5 min using an ultrafiltration centrifuge tube (100 KDa, Millipore). The concentrated solution was centrifuged at 100,000 g for 2 h. After the supernatant was discarded, the purified TExo-N_3_ were obtained in the form of a pellet. The pellet was resuspended in a suitable solution for TEM and Western blot analysis.

**Identification of N_3_ groups on the surface of TExo-N_3_ and IgG Fc on the surface of TExo-Fc**

The DBCO-CY5.5 (Xian Ruixi Biological Technology) solution was added to the purified TExo-N_3_ solution and subsequently shaken at 4 ℃ for 1 h for a bioorthogonal reaction. The unreacted DBCO-CY5.5 was removed by ultracentrifugation, and then the red fluorescence of TExo-N_3_ was detected by nanoflow cytometry (NanoFCM).

The IgG Fc/FITC solution was added to the purified TExo-N_3_ solution and subsequently shaken at 4 ℃ for 1 h for a bioorthogonal reaction. The unreacted IgG Fc/FITC was removed by ultracentrifugation, and then the green fluorescence of TExo-Fc/FITC was detected by nanoflow cytometry (NanoFCM).

**In vivo verification of the metabolic glyco-engineering of 4T1 tumor and the construction of TExo-Fc**

All animal experiments were conducted according to the requirements of the Laboratory Animal Ethics and Welfare Committee of Hebei Medical University (Approval No. IACUC-Hebmu 2024196). 5-week age BALB/c mice were inoculated with 4T1 breast cancer cells in situ. When tumor volumes reach approximately 80 mm^3^, mice randomly divided into two groups: control and Man@Lip. Mice in the Man@Lip group were intratumoral injection of 100 μL of Man@Lip solution. After 24 h, the organs and peripheral blood of the mice were collected. The collected tumor and major organ tissues were used for further immunofluorescence staining. TExo-N_3_ were extracted from mouse major organs and peripheral blood. The extracted TExo-N_3_ were labeled with BDCO-CY5.5 and analyzed for fluorescence intensity by microplate reader.

The 4T1 tumor-bearing mice randomly divided into two groups: control and IgG Fc/FITC-DBCO. Mice in the IgG Fc/FITC-DBCO group were first intratumoral injection of 100 μL of Man@Lip solution, the second intravenous injection of 100 μL of IgG Fc/FITC-DBCO solution over 24 h. After 4 h, the peripheral blood of the mice were collected. TExo-Fc/FITC were extracted from peripheral blood and analyzed for fluorescence intensity by microplate reader.

**The biodistribution of IgG Fc-DBCO**

All animal experiments were conducted according to the requirements of the Laboratory Animal Ethics and Welfare Committee of Hebei Medical University (Approval No. IACUC-Hebmu 2024196). 5-week age BALB/c mice were inoculated with 4T1 breast cancer cells in situ. When tumor volumes reach approximately 80 mm^3^, mice randomly divided into two groups: control and IgG Fc/FITC-DBCO. Mice in the IgG Fc/FITC-DBCO group were intravenous injection of 100 μL of IgG Fc/FITC-DBCO solution. After 6 h, the mice were sacrificed and the main organs were collected. The collected tissues were used for further immunofluorescence analysis.

**Binding kinetics analysis of IgG Fc and FcγRII/III**

FcγRIIB proteins or FcRIII proteins (ACROBiosystems) were conjugated on a CM5 chip. IgG Fc was used as the analyte. The binding changes of the two proteins were determined by a surface plasmon resonance system (Biacore 1K).

**Lung metastasis mode**

All animal experiments were conducted according to the requirements of the Laboratory Animal Ethics and Welfare Committee of Hebei Medical University (Approval No. IACUC-Hebmu 2024196). 5-week age BALB/c nude mice were inoculated with MDA-MB-231 breast cancer cells in situ. When tumor volumes reach approximately 40 mm^3^, mice randomly divided into five groups: PBS, DOX, Man@Lip+IgG Fc-DBCO, DOX+IgG Fc-DBCO and DOX+Man@Lip+IgG Fc-DBCO. The treatment was repeated every 5 days for a total of 3 times. After the end of treatment, 2 × 10^6^ MDA-MB-231 cells were injected into the mice through the tail vein. After 14 days of continuous observation of the survival status of the mice, lung tissues were collected for pathological analysis.

**Statistical Analysis**

GraphPad Prism 8.0 Software was used to perform the statistical analysis. All experiments were performed independently at least three times, and the results were presented as means ± standard deviation. Comparisons between any two groups were performed using the two-tailed, unpaired Student’s t-test. Comparisons among more than two groups were performed using one-way ANOVA. Post-hoc testing was performed using the Turkey HSD method. Single, double, and triple asterisks represent P < 0.05, 0.01, and 0.001, respectively; P < 0.05 was considered statistically significant.


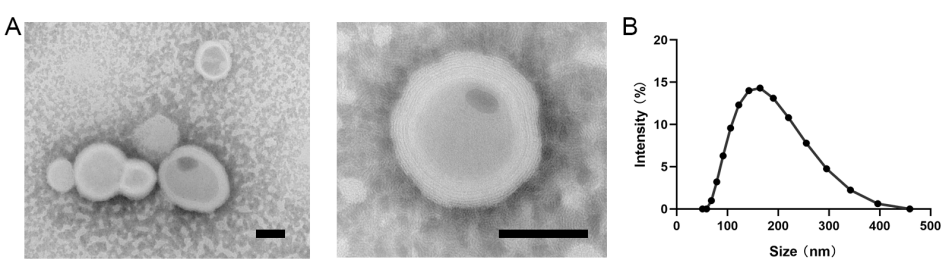


**Figure S1.** Representative TEM image (A) and hydrated particle size (B) result of liposomes. Scale bar, 100 nm.

**
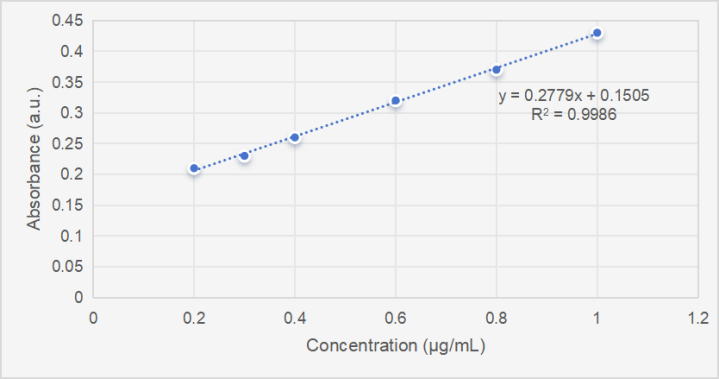
**

**Figure S2.** Standard curve of concentration versus absorbance.

**
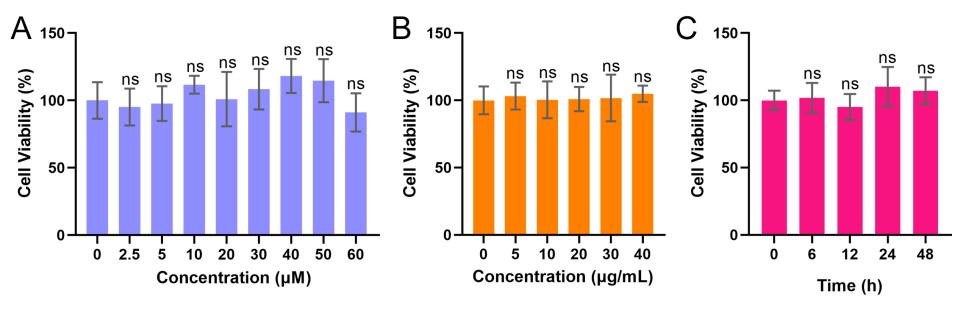
**

**Figure S3.** The effects of mannose-N_3_ (A), liposomes (B) and Man@Lip (C) on the proliferation of 4T1 cells were analyzed by MTT method. Data are presented as the mean ± SD. *p < 0.05; **p < 0.01; ***p < 0.001; ****p < 0.0001.

**
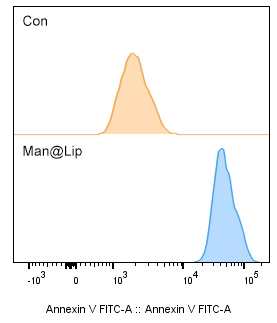
**

**Figure S4.** The flow cytometry result of 4T1 cell uptake of Man@Lip.


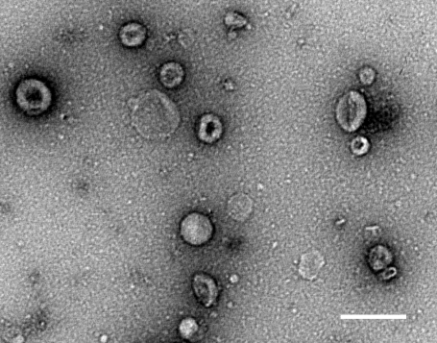


**Figure S5.** Representative TEM image of TExo-N_3_. Scale bar, 200 nm.


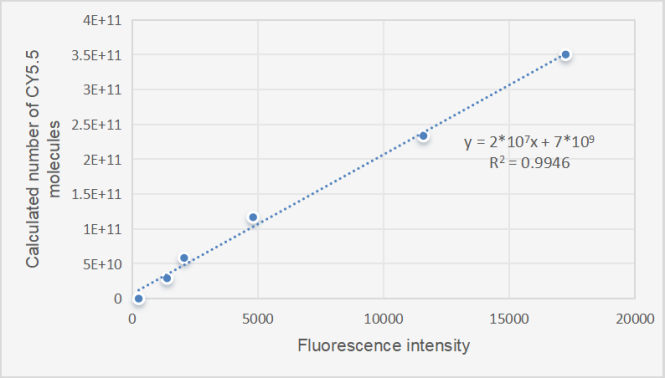


**Figure S6.** Standard curve of fluorescence intensity versus calculated number of CY5.5 molecules.


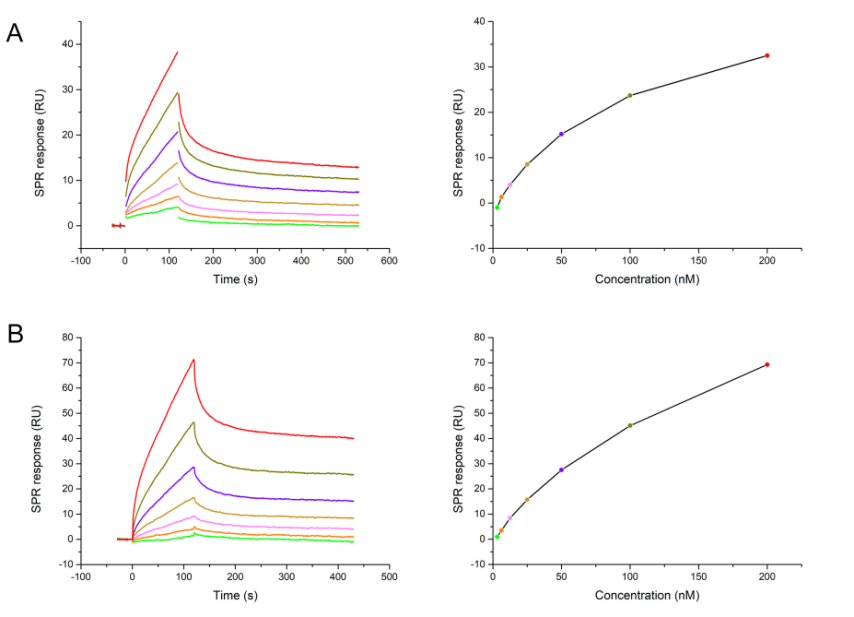


**Figure S7.** The SPR system analyzed the binding of IgG Fc to FcγRIIB protein (A) or FcγRIII protein (B).


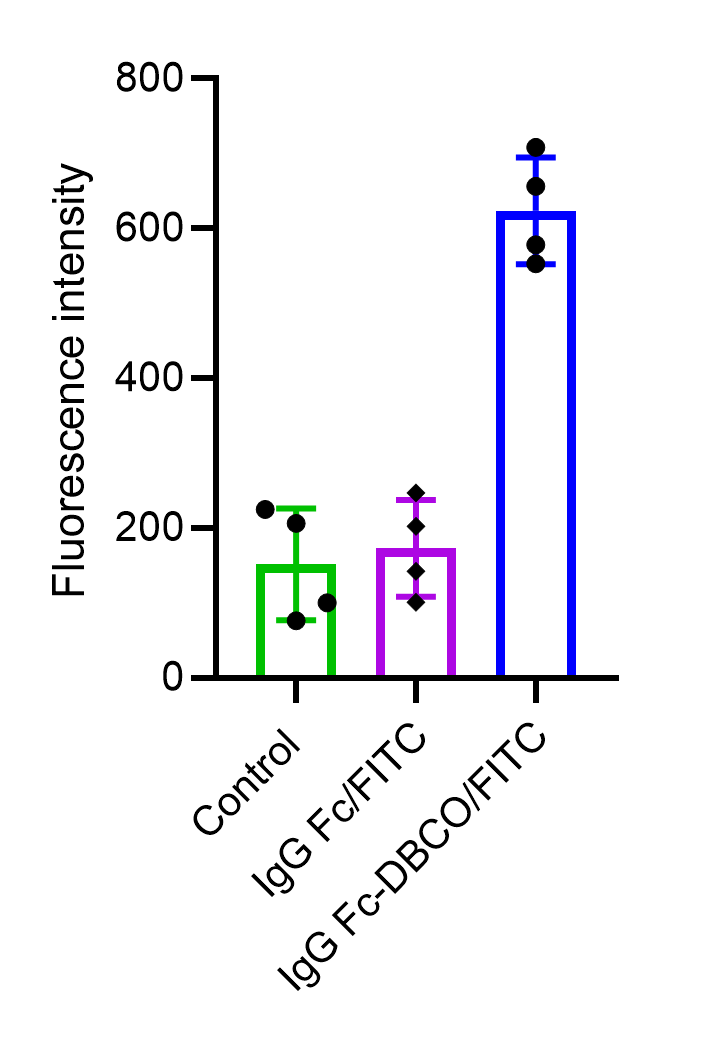


**Figure S8.** Fluorescence intensity statistics of TExo-Fc/FITC in the blood of mice (n=4).


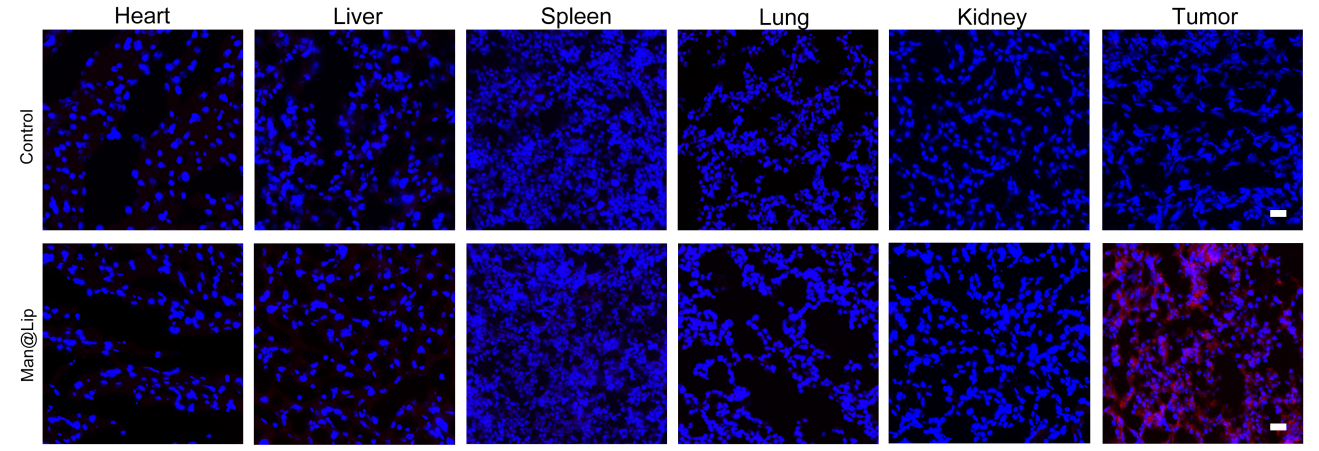


**Figure S9.** Immunofluorescence staining results of major organs after 24h of intratumoral injection of Man@Lip (blue: DAPI; red: DBCO-CY5.5). Scale bar, 20 μm.


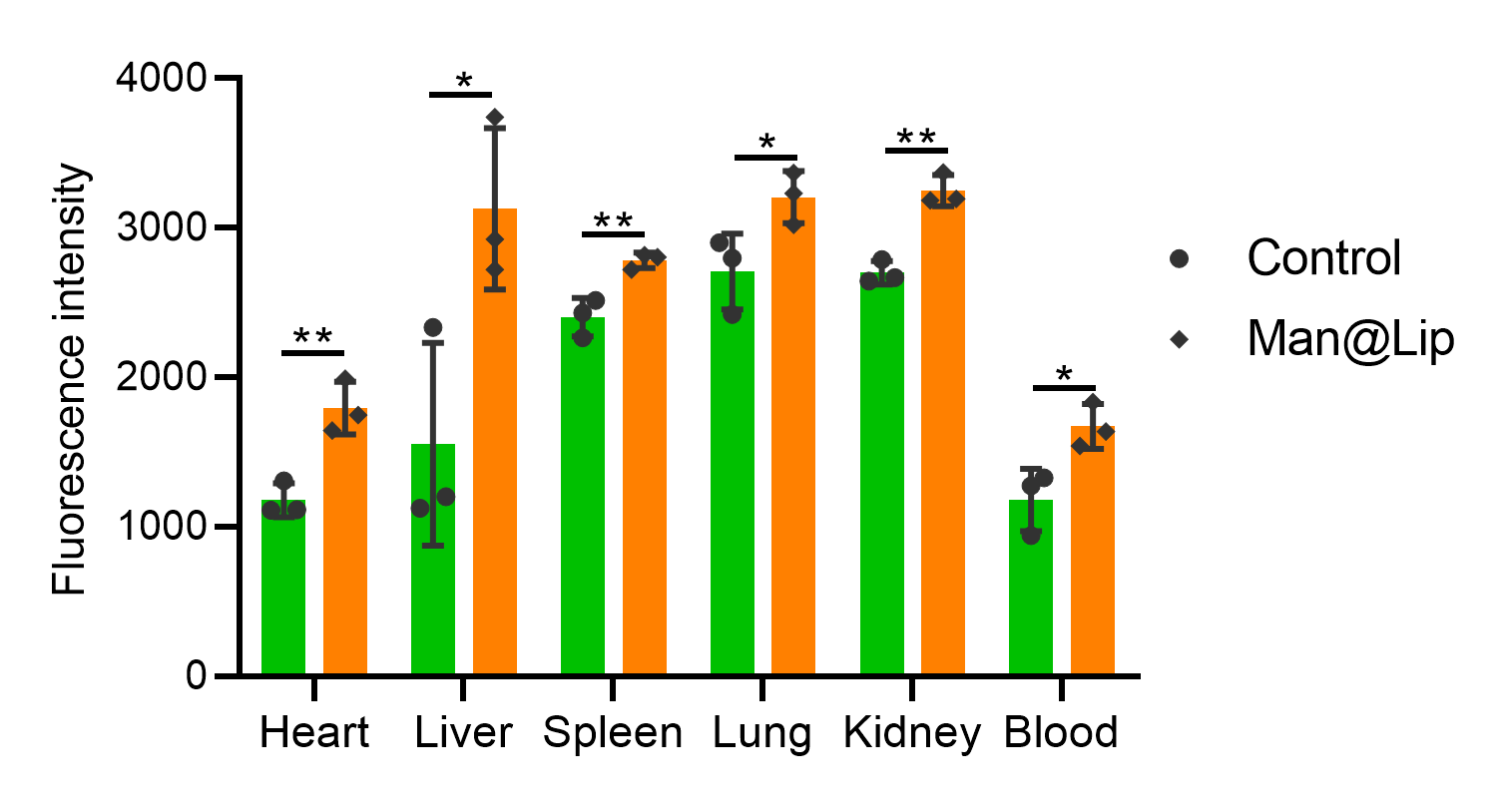


**Figure S10.** Fluorescence intensity statistics of DBCO-CY5.5 labeled TExo-N_3_ in major organs and blood of mice (n=3). Data are presented as the mean ± SD. *p < 0.05; **p < 0.01; ***p < 0.001; ****p < 0.0001.


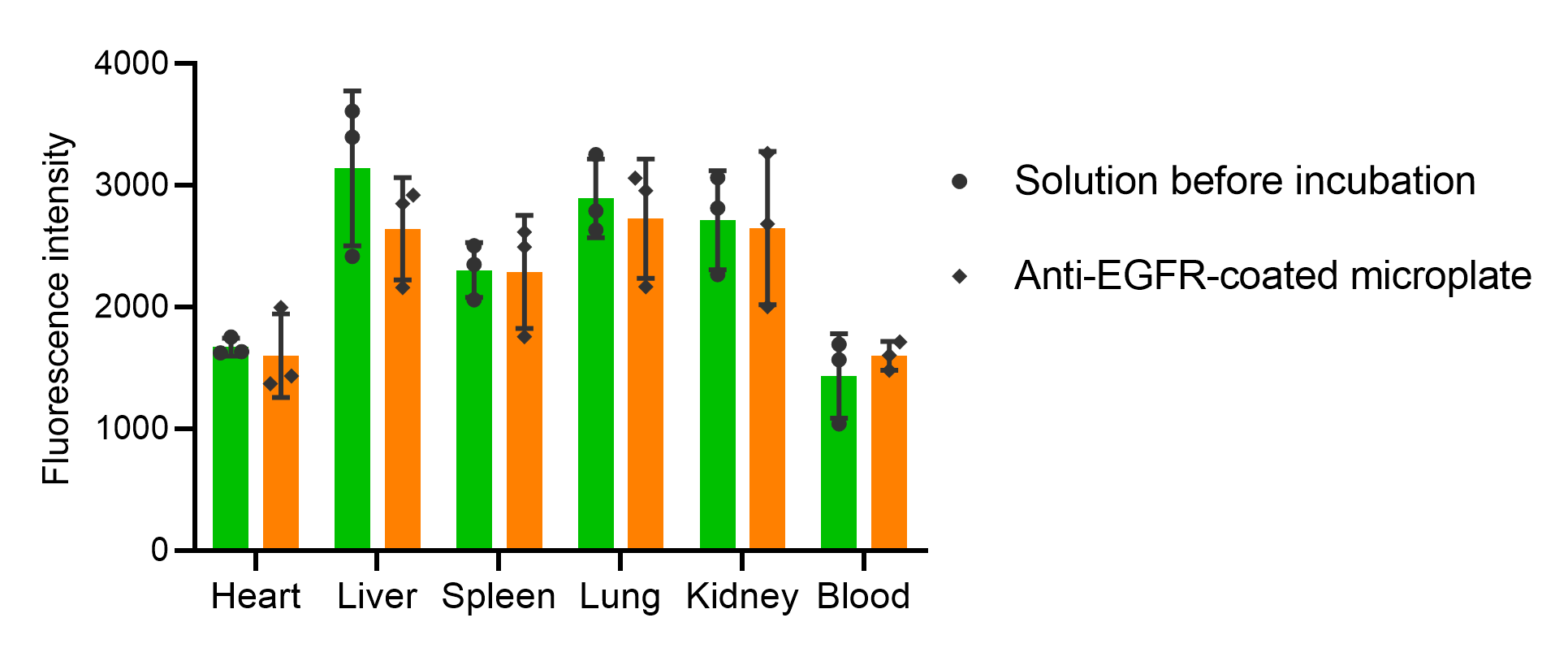


**Figure S11.** Fluorescence intensity statistics of anti-EGFR coated microplates after incubation (n=3).


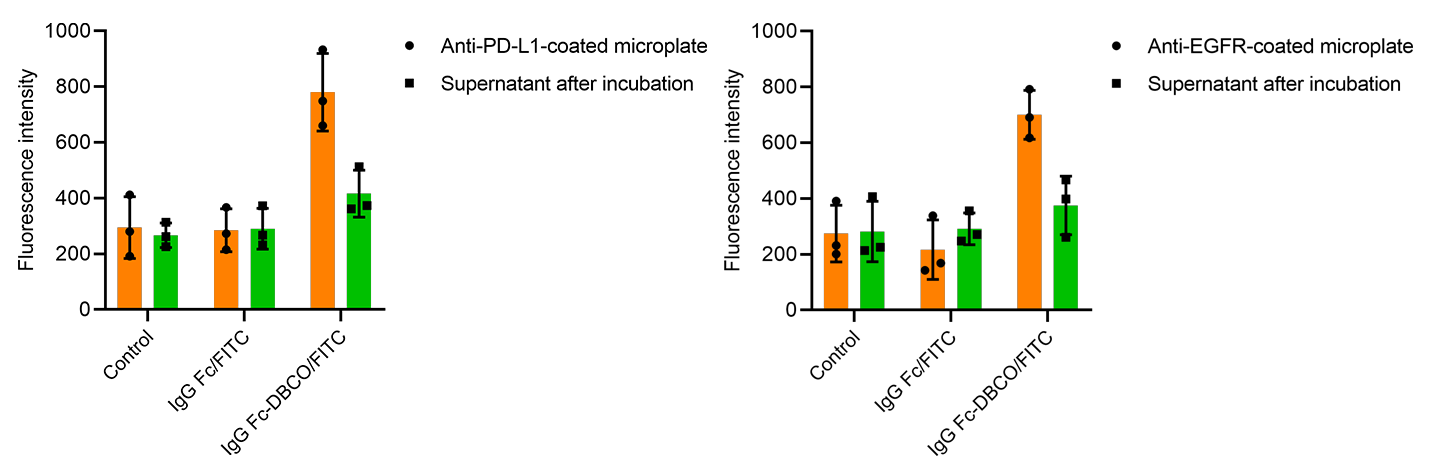


**Figure S12.** Fluorescence intensity statistics of anti-PD-L1 coated or anti-EGFR coated microplates after incubation (n=3).


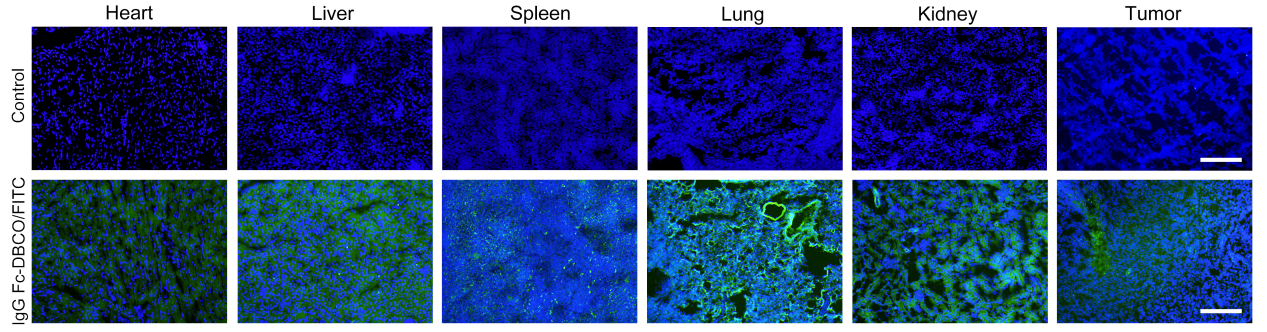


**Figure S13.** Immunofluorescence staining results of major organs after 6 h of intravenous injection of IgG Fc/FITC-DBCO (blue: DAPI; green: IgG Fc/FITC-DBCO). Scale bar, 100 μm.


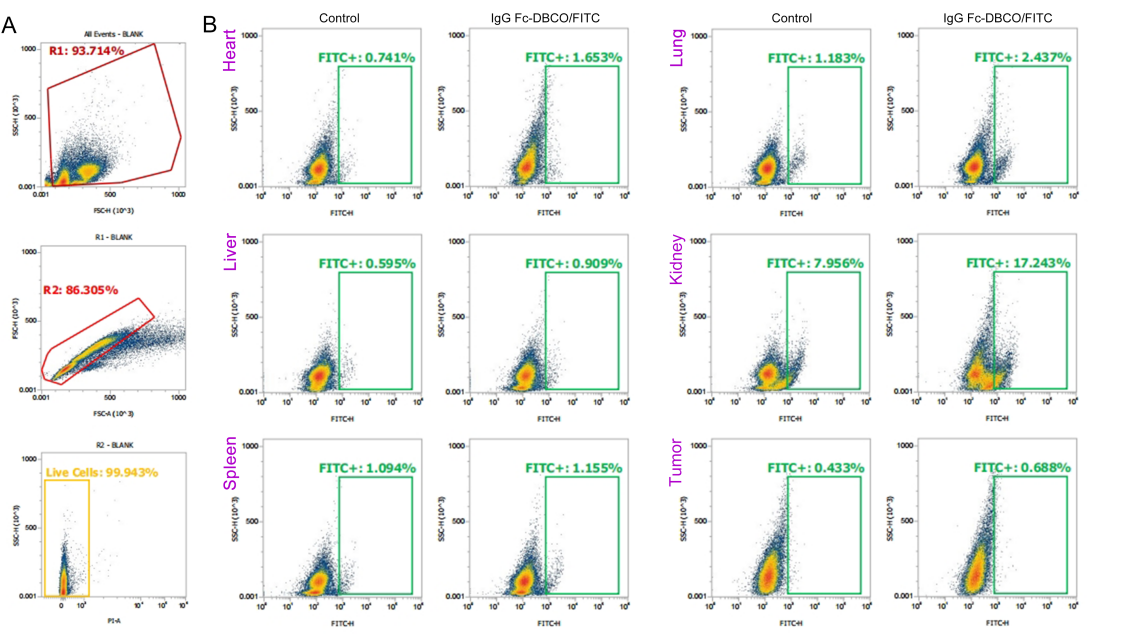


**Figure S14.** Gating strategies and representative results of flow cytometry showed the clearance of TExo-Fc/FITC and free IgG Fc/FITC-DBCO in major organs.


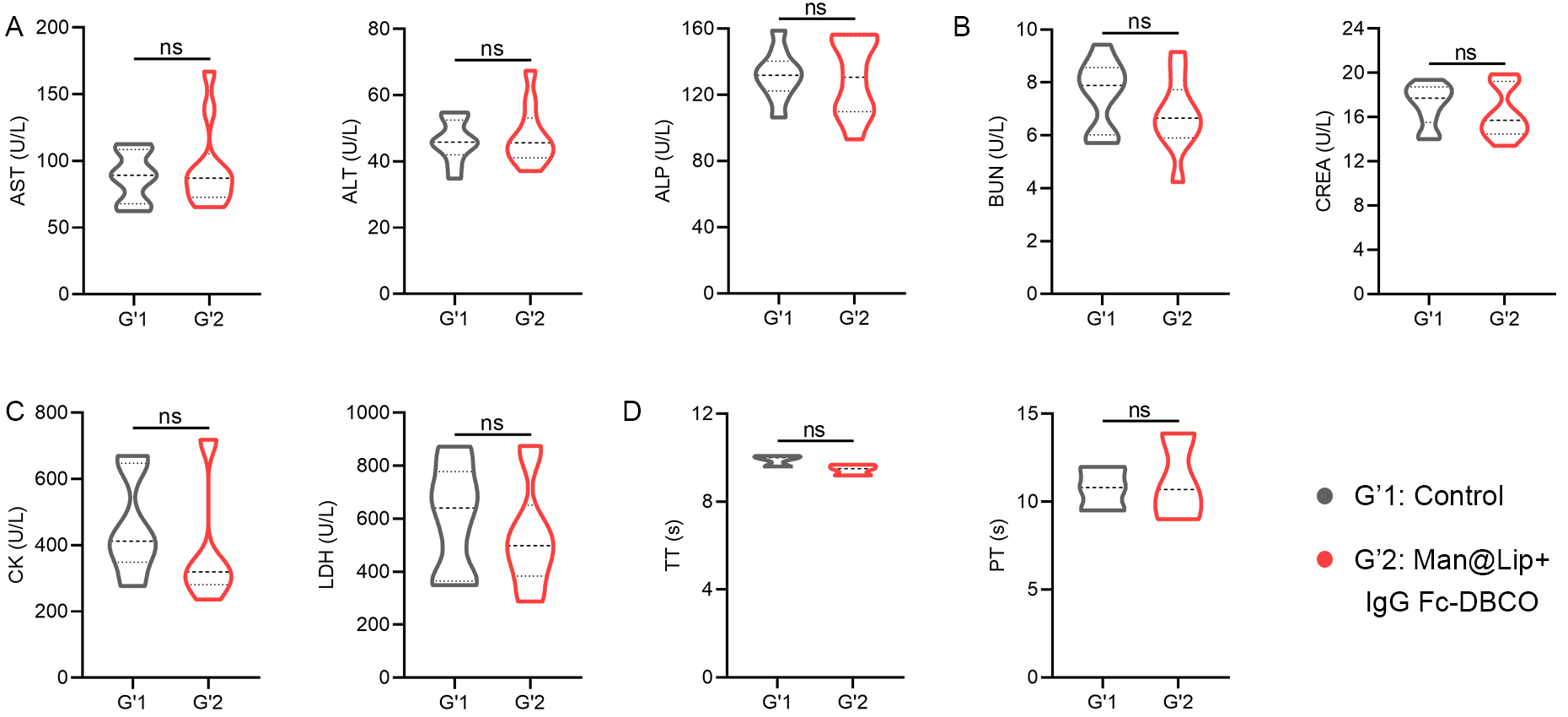


**Figure S15.** (A-C) Blood biochemical results of mice after clearing TExo by Biordee strategy (n=10). (D) Coagulation function assay of mice after clearing TExo by Biordee strategy (n=3). Data are presented as the mean ± SD. *p < 0.05; **p < 0.01; ***p < 0.001; ****p < 0.0001.


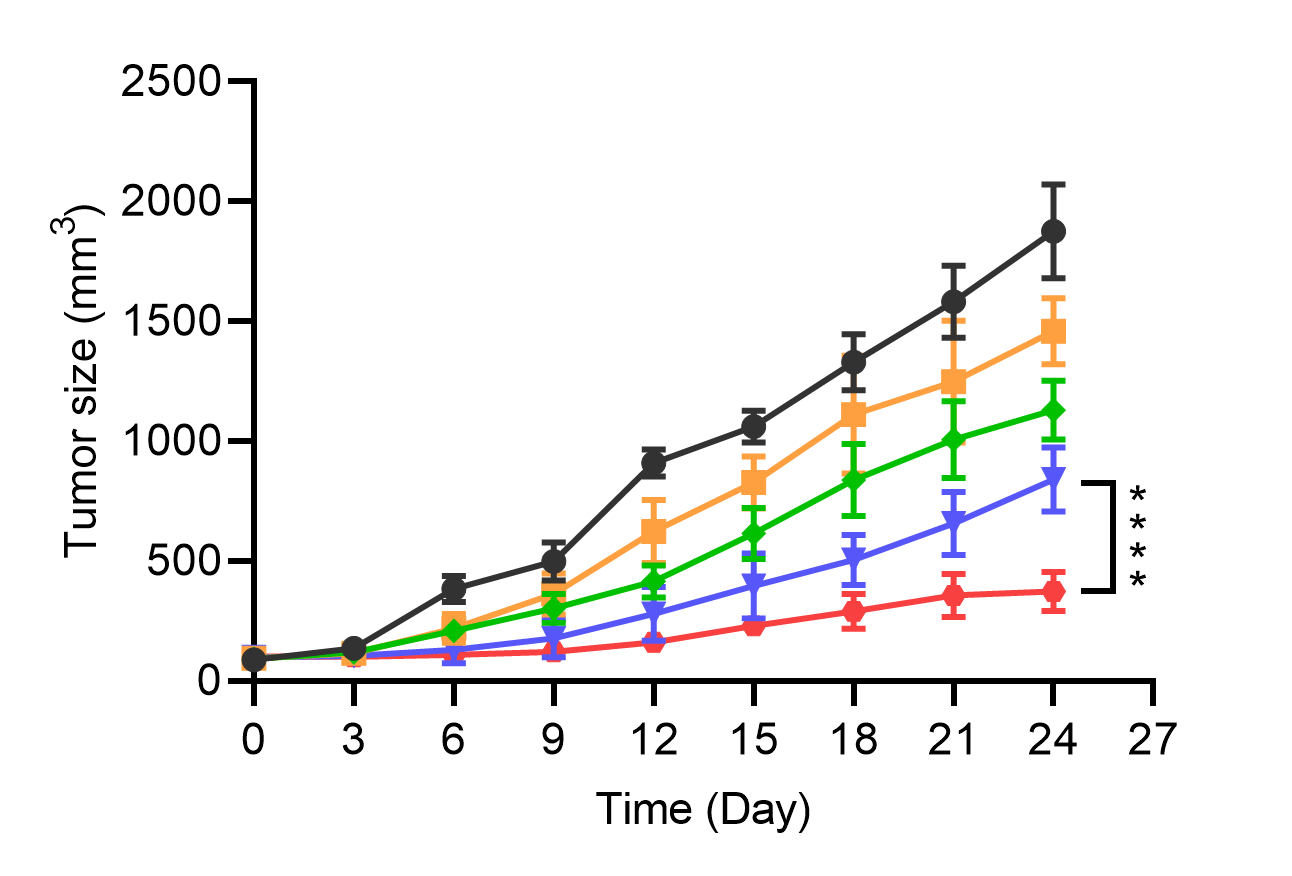


**Figure S16.** Tumor growth curves of mice in different treatment groups. Data are presented as the mean ± SD. *p < 0.05; **p < 0.01; ***p < 0.001; ****p < 0.0001.


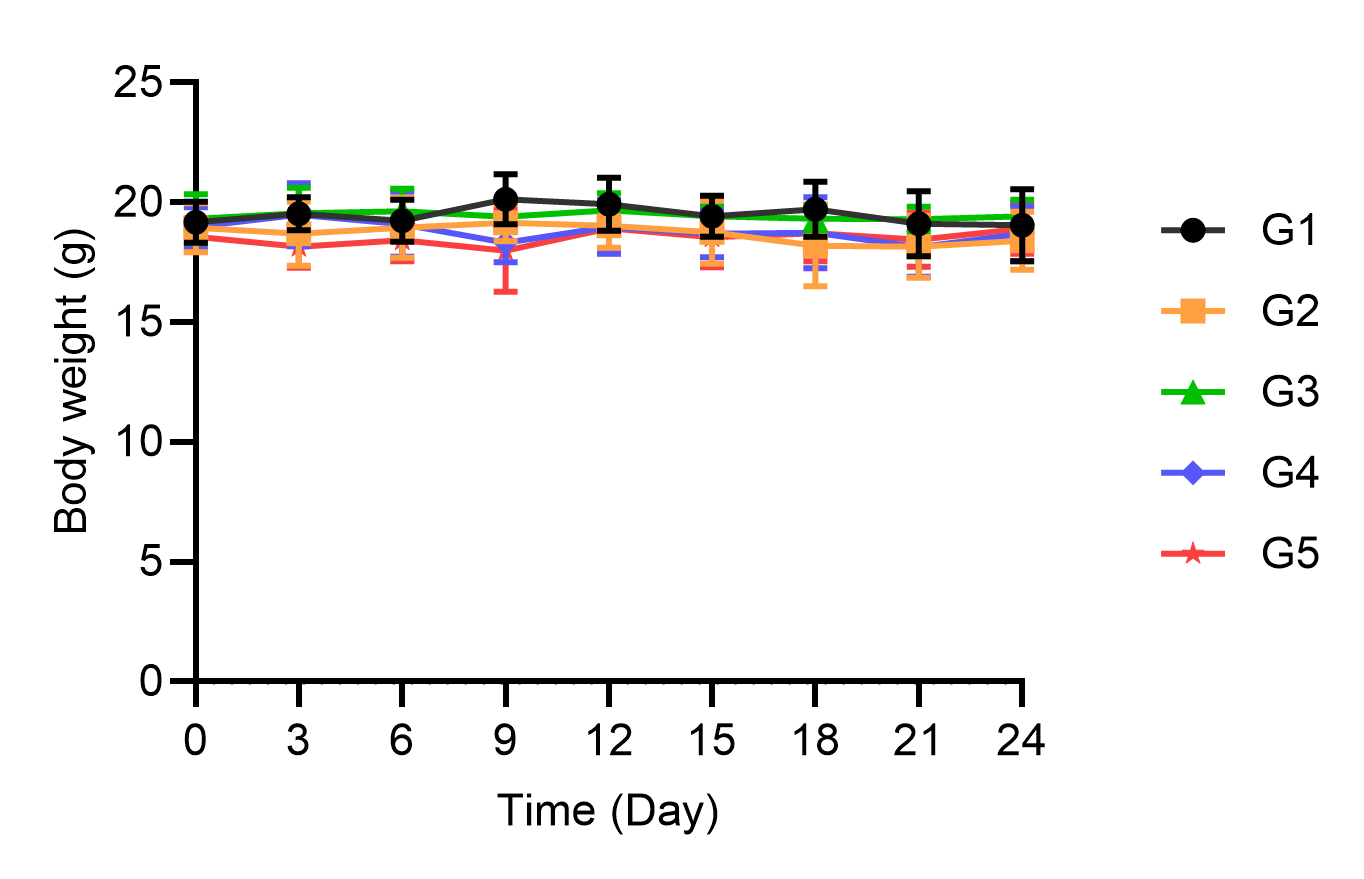


**Figure S17.** Body weight of mice in different treatment groups.


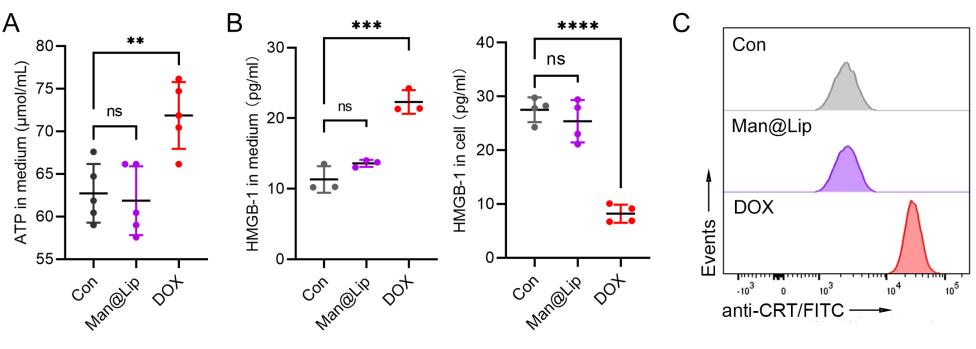


**Figure S18.** The levels of ATP (A), HMGB-1 (B), and CRT (C) changed after treatment of 4T1 cells with Man@Lip and DOX. Data are presented as the mean ± SD. *p < 0.05; **p < 0.01; ***p < 0.001; ****p < 0.0001.

*
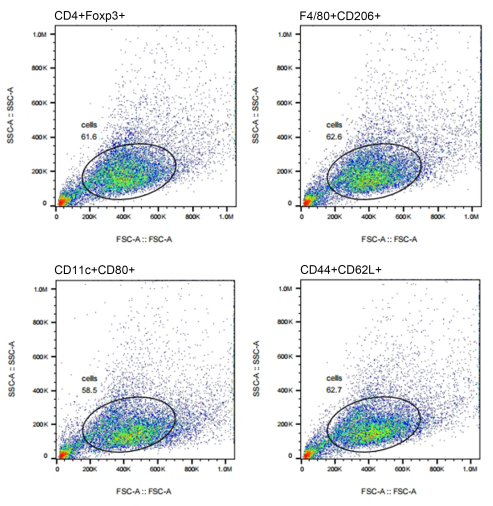
*

**Figure S19.** Gating strategies for flow cytometry in Figure 5B, 5D and 6B.


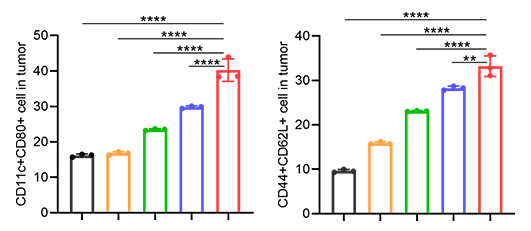


**Figure S20.** Quantitative analysis by flow cytometry showed the proportion of CD11c+CD80+ and CD44+CD62L+ cells in mouse spleen tissues from different treatment groups (n=3). Data are presented as the mean ± SD. *p < 0.05; **p < 0.01; ***p < 0.001; ****p < 0.0001.


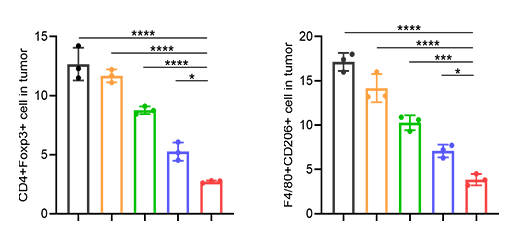


**Figure S21.** Quantitative analysis by flow cytometry showed the proportion of CD4+Foxp3+ and F480+CD206+ cells in tumor tissues (n=3). Data are presented as the mean ± SD. *p < 0.05; **p < 0.01; ***p < 0.001; ****p < 0.0001.


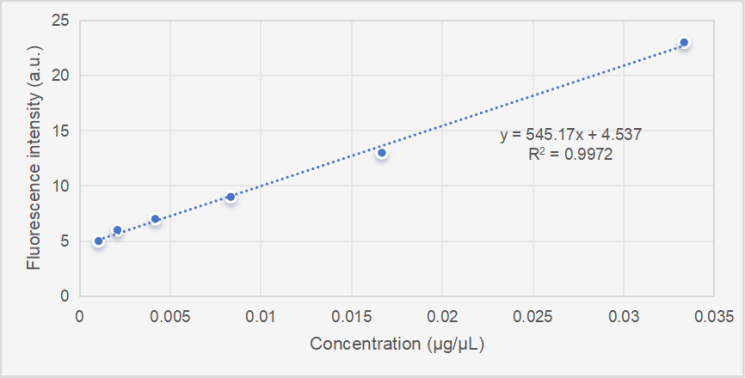


**Figure S22.** Standard curve of concentration versus fluorescence intensity.


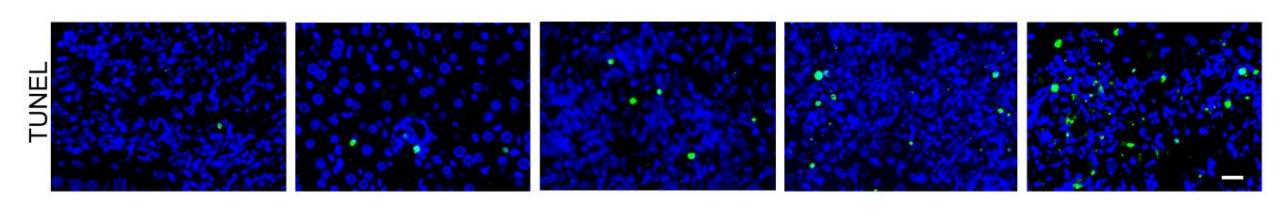


**Figure S23.** TUNEL staining images of liver tissue in different treatment groups. Scale bar, 20 μm.


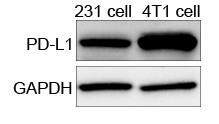


**Figure S24.** Western blot analysis showed PD-L1 expression levels in 231 cell and 4T1 cell.


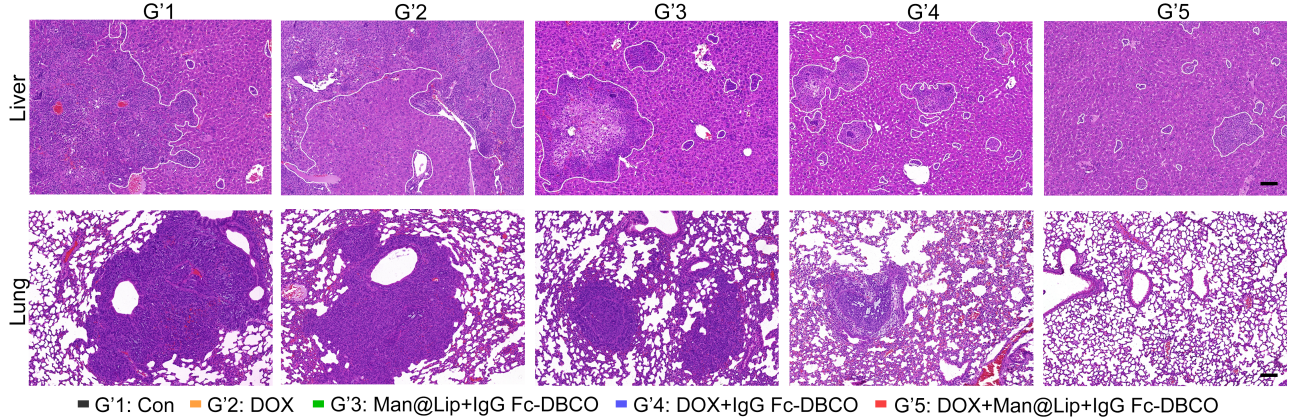


**Figure S25.** H&E staining images of tumor tissue in different treatment groups. Scale bar, 100 μm.
